# The Ambiguous Role of Growth Factors in Autism: What Do We Really Know?

**DOI:** 10.3390/ijms26041607

**Published:** 2025-02-13

**Authors:** Giulia Spoto, Ambra Butera, Maria Ludovica Albertini, Carla Consoli, Graziana Ceraolo, Antonio Gennaro Nicotera, Gabriella Di Rosa

**Affiliations:** 1 Unit of Child Neurology and Psychiatry, Department of Biomedical Sciences, Dental Sciences & Morpho-Functional Imaging, University of Messina, 98125 Messina, Italy; giulia.spoto27@gmail.com (G.S.); gdirosa@unime.it (G.D.R.); 2 Unit of Child Neurology and Psychiatry, Department of Chemical, Biological, Farmaceutical & Environmental Science, University of Messina, 98122 Messina, Italy; butera.ambra@gmail.com; 3 Unit of Child Neurology and Psychiatry, Department of Human Pathology of the Adult and Developmental Age “Gaetano Barresi”, University of Messina, 98125 Messina, Italy; marialudovica.albertini@gmail.com (M.L.A.); carlaconsoli@hotmail.com (C.C.); graziana.c23@hotmail.it (G.C.); 4 Unit of Child Neurology and Psychiatry, Maternal-Infantile Department, University of Messina, 98125 Messina, Italy

**Keywords:** autism spectrum disorder, BDNF, growth factors, IGF1, neurotrophins, NGF

## Abstract

Autism spectrum disorder (ASD) is a complex neurodevelopmental condition with multifactorial origins, including the potential involvement of neurotrophins and growth factors. These molecules, which are crucial for neuronal survival, synaptic plasticity, and brain development, have been implicated in ASD pathophysiology. Altered levels of neurotrophins such as NGF, BDNF, NT3, and NT4, as well as growth factors like IGF1, VEGF, and FGF, have been associated with cognitive deficits, sensory processing abnormalities, and behavioral issues in ASD patients. However, the literature presents conflicting results, often due to differences in research methodologies, sample sizes, patient populations, and diagnostic criteria. Despite these inconsistencies, the potential of neurotrophins and growth factors as biomarkers and therapeutic targets for ASD remains promising. Future research with standardized methodologies, larger cohorts, and a clearer understanding of genetic influences is needed to further elucidate their roles in ASD diagnosis and treatment.

## 1. Introduction

Autism spectrum disorder (ASD) encompasses a group of conditions related to atypical brain development, characterized by impairments in social and communication skills, along with repetitive and restrictive sensory–motor behaviors [1]. Once regarded as a rare disease, it has been recently estimated that 1 in 100 children are diagnosed with ASD globally, with a male-to-female ratio of 4.2 [2].

ASD shows considerably heterogeneous presentations that are now considered a continuum of severity, ranging from very mild to severe [1]. ASD symptoms do not occur in the first year of life but gradually emerge during the second to third year. Therefore, the diagnosis can be made as early as 18–24 months of age, though the average age of diagnosis is not until 4 years or older [2,3]. However, differences in some developmental areas (i.e., motor skills, visual perception, language, and eye gaze patterns of social scenes and faces) have been described during the first year in children at high risk of developing autism or later diagnosed with ASD [3].

This evidence underlies the occurrence of an alteration in neurodevelopment before the unfolding of ASD symptoms and several months before an actual diagnosis can be made. In fact, the neurodevelopmental trajectory is determined by a myriad of biological events that begin very early in life, even before birth, including neuronal processes such as proliferation, migration, synaptogenesis, and myelination [4]. Alterations in genetic, environmental, or both factors may interrupt this complex sequence, potentially leading to neurodevelopmental disorders [5].

Alterations in whole and regional brain volumes, synaptic density, and neuronal networks have been described in autistic patients [6,7]. Previous studies reported brain overgrowth in subjects with ASD, particularly between 12 and 24 months [8,9]. It has been hypothesized that this brain cortex overgrowth and the minicolumnar abnormalities showed by autistic patients could be determined by an excessive production of neural cells [10]. Notably, during the first 2 years of life, the human cortex undergoes an increase in synaptic density, followed at later stages by a targeted elimination of synapses (pruning) that reduces the density of synaptic connections by about 50% [11]. It has been proposed that an increase in the proliferation of neural progenitor cells and hyper-expansion of the cortical surface area may occur in ASD patients during the first year: during this pre-symptomatic period, these children may be affected by disrupted sensorimotor and attentional experiences, leading to altered experience-dependent neuronal development and decreased elimination of neuronal processes [12]. In addition, the impairment in synaptogenesis, namely synaptopathy, or in synaptic pruning may lead to an imbalance in excitatory/inhibitory connectivity, which has a major role in ASD pathogenesis [13,14,15]. For instance, connectivity abnormalities have been reported in children with ASD in the networks of the so-called ‘social brain’, which include the fronto-temporal and fronto-parietal regions [5]. The abnormal activity in specific neural circuits seen in autism could stem from uncontrolled growth of neural connections [10].

**Figure 1:**
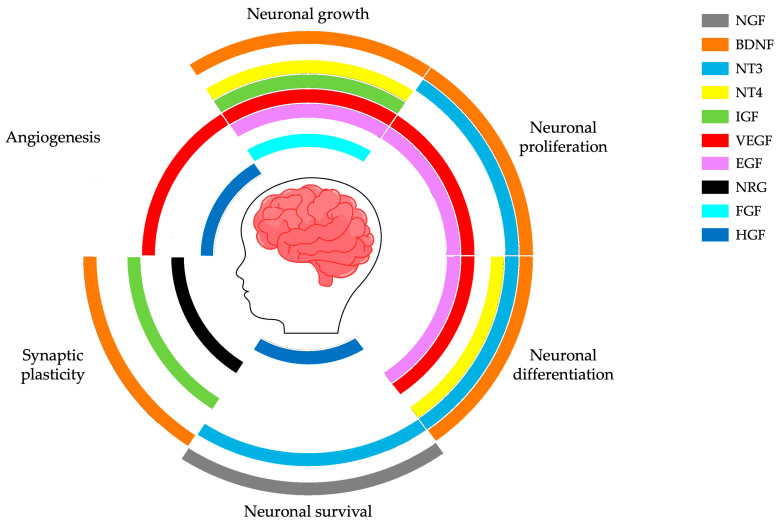
The diagram provides a schematic summary of the role of growth factors in central nervous system development. Disruptions in processes such as neuronal growth, proliferation, differentiation, survival, synaptic plasticity, and angiogenesis may contribute to the etiopathogenesis of neurodevelopmental disorders, including autism spectrum disorder.

All these neurobiological processes that intervene during these early stages of brain development are driven and regulated by numerous biological factors. Among them, growth factors play a crucial part in the modulation of major neurodevelopmental processes (i.e., survival and growth of neurons, synaptic plasticity) that arise during the prenatal and postnatal epochs (see Figure 1) [16].

Their abnormal expression starts during embryogenesis and persists in the later stages of life, contributing to the clinical manifestations of the neurodevelopmental disorder [17]. This evidence supports their role in the development of ASD and could account for the enlargement of the prefrontal and temporal cortices [18]. In this review, we summarize the current knowledge on the most well-known growth factors associated with ASD, exploring their potential role in the pathogenesis of the condition and their possible correlation with autistic symptoms while also considering their potential use as biomarkers for diagnosis and prognosis.

## 2. Neurotrophins

Neurotrophins (NTs) are a group of soluble growth factors with similar structures and functions, identified as key players in neuronal survival during development. They include nerve growth factor (NGF), the first and most well-known NT, brain-derived neurotrophic factor (BDNF), neurotrophin-3 (NT3), and neurotrophin-4 (NT4) [19]. NTs are produced as preproprotein precursors, which are then cleaved intracellularly by metalloproteases to form pro-NTs, which are further processed to generate mature NTs [20]. Two main types of receptors on cell surfaces mediate the genomic effects of NTs: the p75 neurotrophin receptor (p75NTR) and Tropomyosin-related kinase (Trk) receptors [21]. Both receptor types activate distinct signaling pathways. The effects of these receptors are complex, as they can either bind NTs independently or interact with each other to produce different outcomes [22]. p75NTR is a 75 kDa receptor that binds all NTs with low affinity. It belongs to the tumor necrosis factor (TNF) receptor superfamily and, like other family members, contains a death domain that interacts with intracellular adapter proteins. The binding of NTs to p75NTR activates downstream signaling involving transcription factors such as NFκB, which can lead to apoptosis. This process may require the expression of specific adaptor proteins and impaired Trk signaling, resulting from the interaction between p75NTR and the immature forms of NTs. However, its main function is to serve as a coreceptor with Trks to create high-affinity NT receptors [23]. Trk receptors are 140 kDa tyrosine kinases that have a higher affinity for different NTs, especially in their mature forms [24]. Their activity is regulated by p75NTR, which is required for binding NTs with high affinity [23]. TrkA was first identified as the main receptor for NGF, but, in an alternatively spliced form, it can also bind NT3 with different effects. TrkB primarily binds BDNF and NT4, with a lower affinity for NT3, while TrkC preferentially binds NT3 [20,25]. The binding of NTs to Trk receptors triggers the phosphorylation of intracellular tyrosine residues, leading to the activation of downstream signaling pathways such as Ras/MAPK/ERK, Akt, and phospholipase Cγ. These pathways activate transcription factors responsible for neural cell differentiation, survival, apoptosis, and growth [26]. Several studies have implicated dysfunctions in the Ras/MAPK signaling pathway in the pathogenesis of ASD. Notably, research on RASopathies—genetic disorders caused by mutations affecting this pathway—has revealed a high prevalence of ASD-like traits, suggesting a shared neurodevelopmental mechanism. In this context, Adviento et al. conducted a study comparing ASD phenotypes across different RASopathies, including neurofibromatosis type 1, Noonan syndrome, and cardiofaciocutaneous syndrome, and found consistent ASD profiles despite differences in developmental milestones, reinforcing the role of Ras/MAPK pathway disruptions in ASD etiology [27].

### 2.1. Nerve Growth Factor (NGF)

First characterized by Levi Montalcini’s group in 1951, NGF is a pleiotropic molecule that exhibits neurotrophic, metabolotrophic, and immunotrophic effects [28]. The NGF/TrkA interaction, via Raf pathway activation, is crucial for neurite growth and for the development/survival of cholinergic neurons in the central nervous system (CNS), along with the survival and maintenance of peripheral nervous system (PNS) neurons [29]. Additionally, it regulates the concentration of reduced glutathione (GSH) via the MEK/MAP signaling pathway and contributes to mitochondrial function remodeling and the maintenance of cellular energy homeostasis [30,31]. NGF also interacts with the immune system and the inflammatory response, promoting B lymphocyte proliferation and antibody production and increasing the release of mast cell mediators [32]. NGF concentrations are predominantly expressed in brain regions that are highly functional and contain major cholinergic pathways, such as the hippocampus and cerebral cortex. Its expression is modulated by neurotransmitters: specifically, glutamate and acetylcholine lead to an increase while gamma-aminobutyric acid (GABA) results in a reduction in its expression. NGF concentrations in serum and cerebrospinal fluid (CSF) can be elevated in various brain disorders, and this increase is part of the physiological pathways of the neuronal recovery process [28].

Additionally, fluctuations in serum NGF concentrations have been linked to several psychiatric disorders (i.e., anxiety, mood disorders, schizophrenia, and Alzheimer’s disease) and proposed as a marker of clinical severity and progression [30].

To date, data from the literature often report heterogeneous samples (gender, age), non-standardized methodologies (i.e., ELISA kits could not differentiate between pro and mature forms of NGF), and unspecific studies (lack of phenotypic subdivision) that have led to confusing and sometimes conflicting results (see Table 1).

In 2001, Nelson et al. conducted a study on archived newborn blood samples from subjects who developed ASD (*n* = 69), intellectual disability without ASD (*n* = 60), or cerebral palsy (*n* = 63) and from a control group (*n* = 54), proving no significant difference in NGF between the ASD and control children [33].

Conversely, many case–control studies have reported high serum levels of NGF in autistic patients [28,32,36]. Indeed, NGF has been proposed by Dinçel et al. as a serological marker for ASD, particularly in individuals who subsequently developed cognitive deficits, regression, and eventually epilepsy [28].

In line with this study, Gomez-Fernandez et al. divided their sample of 108 subjects (54 ASD patients and 54 controls) into two subgroups: ASD children with neurodevelopmental regression (AMR) and ASD children without neurodevelopmental regression (ANMR). The ANMR group showed higher NGF concentrations than the controls [35]. Some authors have focused their attention on a possible autoimmune disorder as the basis of some forms of autism, supported by the activity of brain-specific autoantibodies. Additionally, they studied serotonergic pathways as alterations in serotonin levels could result in abnormalities in brain function and behavior. Mostafa et al. conducted a case–control study on 44 subjects (22 children with ASD and 22 controls) in which they analyzed the relationship between plasma levels of NGF, hyperserotonemia, and the frequency of serum anti-myelin basic protein (anti-MBP) autoantibodies. Levels of NGF, serotonin, and anti-MBP were significantly higher in patients with ASD than in the controls. There was a significant positive correlation between NGF and serotonin levels in ASD patients. In conclusion, serum NGF levels were elevated and significantly correlated with the hyperserotonemia found in many autistic children [32]. Other studies have also evaluated and compared the effectiveness of different interventions on NGF levels in children with autism. Indeed, Moradi et al. observed that NGF levels were globally increased in their examined patients. In particular, the group treated with vitamin D supplementation showed a significant increase in NGF levels compared to the group treated with perceptual activity and the control group, while the group treated with perceptual–motor activities and vitamin D supplementation showed the highest NGF levels. In consideration of this evidence, they suggested a combined approach that includes perceptual–motor activities and vitamin D supplementation for this category of patients to maximize the neuroprotective effects of NGF in the nervous system of these subjects [36].

Additionally, some studies analyzed the presence of NGF receptors, TrkA and p75^NTR^, in the inner mitochondrial membrane, hypothesizing a possible role in mitochondrial biogenesis. In particular, Gevezova et al. hypothesized that NGF-induced neuronal development requires large amounts of energy, and mitochondria in ASD appear to be overloaded to meet the new functional necessities. To test this hypothesis, they conducted a case–control study examining protein levels of NGF in parallel with mitochondrial activity in 52 subjects (40 ASD patients and 12 healthy children). They found an action on the modulation of mitochondrial activity made by NGF that may be important for neuroregeneration or neurogenesis, although it is not clear yet whether these changes are a result or a consequence of ASD [31].

### 2.2. Brain-Derived Neurotrophic Factor (BDNF)

BDNF is synthesized by both neuronal and non-neuronal cells, including endothelial cells, astrocytes, and oligodendrocytes. It is noteworthy that BDNF levels in cerebral endothelial cells are nearly 50 times greater than those observed in cortical neurons [20,37]. BDNF is widely expressed in the CNS, and during the fetal stage, it represents a key regulator of brain development and maturation, contributing to neuronal growth, differentiation, and both synaptic and structural plasticity [38]. Moreover, BDNF is also involved in promoting neurogenesis, maintaining brain homeostasis, and protecting the brain from the neurotoxic effects of inflammation, and, alongside NT3, it regulates angiogenesis and supports vessel maintenance during embryogenesis [38,39,40]. During later stages, BDNF plays a critical role in long-term potentiation by upregulating N-methyl-D-aspartate (NMDA) receptors, inducing the expression of GABA receptor subunit genes, and promoting the synthesis of choline acetyltransferase, thereby facilitating the production and release of acetylcholine [38,41]. Reduced BDNF/TrkB signaling has been linked to impaired synaptic plasticity in hippocampal GABAergic neurons, a mechanism thought to contribute to the development of ASD [42].

Numerous studies analyzed the effects of altered BDNF levels in patients with neuropsychiatric disorders and especially ASD (see Table 2), although the results on the correlation with ASD were inconclusive [33,34,35,43,44,45,46,47,48,49,50,51,52,53,54,55,56,57,58,59,60,61,62,63,64,65,66,67,68,69,70,71,72,73,74].

In fact, comparing ASD patients with typically developed subjects, some studies found a statistically significant increase in BDNF levels [33,43,44,49,53,55,58,59,60,61,62,65,66,68,69,70], while some other authors described statistically significant reduced levels of BDNF in ASD [46,50,52,54,57,64,67,72,73] or no significant differences [34,35,45,48,51,56,72,74]. The observed discrepancies in the results across studies may be attributed to variations in the sample size, as most studies involved small cohorts of patients. Additionally, differences in methodologies and the types of biological specimens utilized could further contribute to these inconsistencies, as the studies employed different biological samples, including serum, plasma, whole blood, platelets, dried blood spots, and brain tissue homogenates. In particular, platelets represent the major peripheral source of BDNF, containing BDNF levels 100–1000 times higher than those found in neurons [73]. In this light, Correia et al. analyzed BDNF levels in platelet-rich plasma and Carpita et al. in platelets [49,73]. On the contrary, some authors considered the amounts of BDNF deriving from platelets as confounding and not clinically relevant; therefore, they analyzed the BDNF levels in platelet-poor plasma [59] or in platelet-free plasma [52]. Similarly, Farmer et al. evaluated BDNF levels while controlling for the platelet count [70]. Age could be considered another confounding factor, as Katoh-Semba et al. observed age-related changes in BDNF levels in the sera of 56 ASD patients vs. 218 control subjects. The authors proved that BDNF levels gradually decreased in the control subjects with a small peak between 30 and 39 years. Conversely, the ASD subjects exhibited an additional peak in BDNF levels between 10 and 19 years, presenting the lowest BDNF levels at 0–9 years of age [47]. Nevertheless, subsequent studies did not confirm a significant correlation between BDNF levels and age [49,60]. It is also worth noting that increased levels of BDNF have been reported in patients with intellectual disability (ID), which is the main comorbidity of ASD [33,43]. Ormstad et al. reported a statistically significant increase in BDNF levels in autistic patients with ID vs. high-functioning ASD patients [65]. Very recently, Cui et al. compared 67 ASD subjects with 47 subjects with ID, proving no statistically significant difference in BDNF levels [74]. In contrast, Taurines et al. found a non-significant trend of increasing BDNF levels in autistic patients who also presented with attention deficit hyperactivity disorder (ADHD) in comparison to ASD subjects without ADHD [57]. A significant correlation of BDNF levels with hyperactivity in ASD children was demonstrated by Spratt et al., but only in the female subjects [63]. Inconsistent findings were also reported regarding the association between BDNF levels and autistic symptoms. Zhang et al., Wang et al., Meng et al., and Cui et al. observed increased BDNF levels in autistic subjects that were statistically significantly associated with ASD symptoms measured with the Childhood Autism Rating Scale (CARS) [58,61,62]. Similar results were reported by Ricci et al., though the correlation did not reach statistical significance [55]. An association between BDNF levels and autistic traits was also reported by Bondino et al. in the general population [75]. However, the opposite results were observed by Correia et al. and by Francis et al., who found no correlation with ASD symptoms [49,64]. In addition, Han et al. revealed a negative significant correlation between BDNF levels and autistic symptoms [72].

### 2.3. Neurotrophin-3 (NT3)

NT3 was the third neurotrophin to be discovered and was originally called hippocampus-derived neurotrophic factor, given its high mRNA expression in the hippocampus [76]. It plays an important role during development in regulating the proliferation, differentiation, and survival of different neuronal populations, but in adulthood, NT3 has a limited functional role, regulating the maintenance and survival of more selected neuronal populations [77].

Despite their importance, little is known about the role of NTs in childhood, and the data regarding NT3 and its role in the etiopathogenesis of ASD remain limited (see Table 3) [78].

Nelson et al. conducted a case–control study in which a reduction in the serum NT3 concentration was observed in autistic patients compared to healthy controls. In particular, the NT3 concentration was lower in adults than in newborns [45]. Another study examined the concentration of NT3 and other neuropeptides, cytokines, and nitric oxide (NO) in plasma, observing a lower concentration of NT3 and a higher plasma concentration of Vasoactive Intestinal Peptide (VIP) and Interferon-Gamma (IFN-γ) in ASD patients compared to healthy subjects, suggesting a blockage of the VIP-induced production of trophic factors, including NT3 [78]. Sajdel-Sulkowska et al. conducted a study on human postmortem frozen cerebellar tissue from control and autistic donors, finding an increase in NT3 levels in the cerebellum of ASD subjects compared to healthy controls. These data suggest that altered levels of NT3 in the brain may contribute to autistic pathology by affecting synaptic plasticity, enhancing oxidative stress, and contributing to Purkinje cell abnormalities [79]. None of the studies examined the possible relation between NT3 levels and autistic symptoms.

### 2.4. Neurotrophin-4 (NT4)

The effects attributed to NT4 are numerous and complex: Embryonic studies have shown that NT4 plays a protective role for sensory neurons and neural crest placodes, promotes the differentiation of motor neurons from basal forebrain neurons, and stimulates dendritic growth in some in vitro studies. On the other hand, NT4 also seems to be associated with pro-apoptotic processes [81].

The role of NT4 in ASD remains uncertain, with changes in mRNA levels not always reflecting protein levels [33,43]. Age differences and the varying expression of neurotrophins may account for these discrepancies. Indeed, the available data from the literature are conflicting; although some studies have shown elevated levels of the NT4 protein in the plasma, serum, and whole blood of autistic individuals [33,43], others have reported lower levels [80], and the data did not always yield significant results (see Table 4) [16,34,82].

Segura et al. [80] examined the gene expression of neurotrophins, including NT4, in peripheral blood samples of patients with ASD. The main goal was to clarify the role of NT-related genes in the pathophysiology of ASD, assess their relevance as potential disease markers, and study their correlation with typical autism characteristics. *NT4* gene expression was found to be significantly lower in patients with ASD compared to healthy controls. The variation in *NT4* gene expression was less marked but still significant, with higher values in controls than in cases [80]. Abdallah et al. also examined NT4 levels in the neonatal dried blood spot samples of 359 ASD vs. 741 frequency-matched controls, reporting statistically significant reduced levels in the ASD patients [54]. Finally, the same authors conducted a case–control study on the amniotic fluid of 414 ASD patients matched with 820 controls. They analyzed the levels of certain matrix metalloproteinases, neurotrophins (BDNF, NT4), and transforming growth factor β (TGFβ) in 414 individuals later diagnosed with ASD and 820 controls. For NT4, over 90% of the measurements were below the working ranges (the concentration range in which the coefficient of variation was below 25%), and no statistically significant values were obtained for BDNF levels [82].

## 3. Other Growth Factors

Increasing evidence indicates that growth factors modulate motor, emotional, and cognitive functions, which may explain various clinical manifestations of psychiatric disorders. Furthermore, alterations in the expression levels of growth factors during embryogenesis are linked to the pathophysiology and clinical manifestations of various neurodevelopmental disorders, including ASD [10].

Abnormal levels of several growth factors have been demonstrated in adults with ASD, including insulin-like growth factor (IGF), vascular endothelial growth factor (VEGF), epidermal growth factor (EGF) and neuregulins (NRGs), fibroblast growth factor (FGF), and hepatocyte growth factor (HGF), which appear to play an important role in neurological development and immune function [83,84,85].

### 3.1. Insulin-like Growth Factor (IGF)

The insulin-like growth factor (IGF) system comprises two hormones: IGF1 and IGF2. These hormones are structurally related to insulin and act through specific tyrosine kinase receptors, IGF1R and IGF2R. IGF1 is a crucial regulator of fetal growth and the development of most organs, especially the CNS. During gestation, IGF1 is secreted by placenta, stimulating the placental transfer of essential nutrients from the mother to the fetus, and its concentrations are positively associated with fetal size and fat mass [86]. However, throughout fetal brain development, IGF1 expression undergoes changes. Initially abundant in many brain areas, it is later confined to a few regions and is maintained at very low levels once the brain is fully formed [86]. Similarly, CNS sensitivity to IGF1 varies among different regions. For instance, cerebellar Purkinje cells, granule cells, oligodendrocytes, and motor neurons are particularly sensitive to IGF1 deficiency, while other cells are independent of the presence of IGF1 [83].

In postnatal life, IGF1 has a systemic expression, primarily produced in the liver under the regulation of growth hormone (GH) secreted by the pituitary gland, and circulates bound to insulin-like growth factor-binding proteins (IGFBPs) as a complex, which regulates its bioavailability, localization, and activity [83,87,88]. Meanwhile, a residual amount of IGF1 is also produced locally in the brain [88]. Systemic IGF1 can cross both the blood–brain barrier and the blood–cerebrospinal fluid barrier, impacting on early CNS development and neuronal plasticity. This suggests that IGF1 acts as an endocrine, paracrine, and autocrine hormone [89]. Once bound to the IGF1 receptor, IGF1 activates the mitogen-activated protein kinase/extracellular signal-regulated kinase (MAPK/ERK) and phosphatidylinositol-3 kinase/mammalian target of rapamycin/serine-threonin-specific protein kinase (AKT-PKB PI3K/AKT1/mTOR) pathways, which are implicated in the processes of brain cell proliferation, neurogenesis, myelination, maturation, and differentiation and prevent apoptosis [71]. In fact, IGF1 enhances the secretion of various neurotransmitters and mediates the effects of other neurotrophic factors (i.e., BDNF and VEGF) [83,87].

Given its critical neuroprotective role, alterations in IGF concentrations have long been extensively studied regarding their potential impact on the pathogenesis of autism spectrum disorder. Several studies in the literature compared autistic patients to neurotypical subjects, measuring IGF1 levels in the brain parenchyma, CSF, plasma, serum, and urine. The findings have been inconsistent and mixed, likely due to the small study populations and varying methodologies across different studies (see Table 5).

Mills et al. detected significantly higher plasma IGF1 levels in children with ASD compared to controls and found a strong correlation with larger head circumference [93]. Another case–control study conducted by Şimşek et al. on 40 subjects (37 males and 3 females, aged 4–12 years) with ASD and 40 healthy children showed a significant increase in serum IGF1 levels among young boys and girls with mild to moderate autism. However, they found no correlation between increased serum IGF1 levels and sociodemographic or clinical scale scores. Accordingly, considering the crucial role of IGF1 in myelination and cell survival, they justified this result of high serum IGF1 levels in the ASD group with a compensatory mechanism for white matter and myelin abnormalities [84].

Similarly, Robinson-Agramonte et al. found a significant positive correlation between serum IGF1 levels and CARS scores in a sample of 22 children with ASD compared to 29 controls. It should be noted that the authors also reported reduced excretion of IGF1 in the urine of young subjects with ASD. This decreased excretion might be due to the increased binding of IGF1 by IGFBP-3, which is elevated in the blood of autism patients, potentially influencing the serum concentration of IGF1 [71]. Another study conducted by Vargas et al. in 2005 on brain parenchyma observed no significant difference in IGF1 levels between the ASD and control groups in terms of the middle frontal gyrus and cerebellum but increased IGF1 levels in the anterior cingulate gyrus in ASD cases [91]. A recent postmortem study by Cioana et al. found no significant differences in IGF1 protein or IGF1 mRNA levels in fusiform gyrus tissues between the ASD and control groups. This suggests that IGF1 or its receptor levels in the brain may not be dysregulated in individuals with ASD [95].

Vanhala et al. found lower CSF IGF1 concentrations in ASD patients compared to a control group. However, the latter included children with various neurological diseases and complaints, introducing a bias in the results [90]. Another study conducted by Riikonen et al. in 2006 on CSF found lower levels with a significant negative correlation between IGF1 and head circumference. According to the authors, low concentrations of IGF1 might indicate that this crucial survival factor is insufficient during the critical brain spurt period, potentially affecting normal Purkinje cell development [92]. Some authors also attempted to measure IGF1 in urine samples, finding significantly lower urinary IGF1 levels in individuals with ASD compared to controls, but without a significant correlation [94].

Based on these findings, some authors proposed IGF1 as a potential tool for ASD treatment. In particular, Marchetto et al. explored how neural cells derived from autistic patients show differences compared to controls, focusing on variations in cell proliferation and neural networks. They investigated the potential therapeutic effects of IGF1 on these altered neural cells. Treatment with IGF1 in neurons derived from ASD patients partially rescued deficits in neuronal networks, as evidenced by improvements in the neuronal spike number and activity [98].

### 3.2. Vascular Endothelial Growth Factor (VEGF)

VEGF is a potent growth factor that plays a crucial role in vasculogenesis, angiogenesis, and the regulation of vascular function [99]. The VEGF family comprises VEGF-B, VEGF-C, VEGF-D, VEGF-E, VEGF-F, placental growth factor, and VEGF-A, which is the most studied [100]. The gene encoding VEGF-A is located on chromosome 6p21.5 and, through alternative splicing, encodes for multiple isoforms, including VEGF-A 121, VEGF-A 145, and VEGF-A 165 [101,102]. VEGF-A 165 is the predominant form in the CNS and exhibits direct trophic and neuroprotective effects on various types of neural cells, including motoneurons, astroglia, microglia, muscle satellite cells, and various neurons (hippocampal, dopaminergic, cortical, cerebellar, and sympathetic neurons) [101,103,104,105,106]. Indeed, in addition to its established roles in vasculogenesis, angiogenesis, and the regulation of vascular function, VEGF is involved in several aspects of brain development: influencing the growth, proliferation, and differentiation of neurons, providing neuroprotection, stimulating neurogenesis, impacting synaptic transmission, and playing a role in the growth and proliferation of glial cells [84].

To date, various studies have reported a relationship between VEGF and ASD, although the results have often been heterogeneous and inconsistent, suggesting that serum or plasma VEGF levels may not be reliable predictors of ASD diagnosis (see Table 6).

Additionally, it remains unclear whether serum VEGF concentrations correspond to VEGF levels in the CNS [84]. Emanuele et al. conducted a case–control study measuring VEGF and its soluble receptors sVEGFR-1 and -2 in adult patients with severe ASD (*n* = 22) and healthy subjects (*n* = 28). They found lower VEGF and increased sVEGFR-1 serum concentrations in patients with ASD compared to controls, suggesting that the decrease in serum VEGF levels could contribute to neuronal loss and a regional reduction in hippocampal size [107]. However, other studies did not show significant differences in serum VEGF levels between ASD subjects and controls [84,108]. Zakareia et al. examined the concentration of VEGF in plasma, but did not find significant differences between cases and controls [109].

### 3.3. Epidermal Growth Factor (EGF)

EGF is a growth factor that precisely stimulates growth, proliferation, and cellular differentiation by binding to its receptor EGFR. It can be detected in all regions of the adult and developing brain, as well as in most developing neurons and astrocytes [112]. EGF rapidly crosses the blood–brain barrier, suggesting that peripheral levels of EGF could be representative of those in the central nervous system [113].

Several studies have analyzed plasma levels of EGF in children with ASD, yielding varying results (see Table 7).

For instance, Russo et al. designed a study to determine plasma EGF levels in a group of children with ASD and correlate these levels with clinical severity (assessed using the Pfeiffer Questionnaire) and other biomarkers. It was found that plasma EGF levels in children with ASD were significantly lower than in neurotypical controls [113]. Similar results were obtained by Suzuki et al., who measured EGF levels in a sample of adults, finding that serum EGF levels in high-functioning autism subjects were significantly lower than those of normal subjects. No significant correlations were found between serum EGF levels and clinical variables [114]. Additionally, a negative correlation was found with hyperactivity, general motor skills, and digitigrade walking, as well as a relationship between decreased EGF and disease severity, and an association between EGF and increased inflammation (increased GABA), except in autistic children with gastrointestinal inflammatory disease [113]. Manzardo et al. also confirmed this result, further demonstrating that EGF is involved in wound healing, thus suggesting that the decrease in children with ASD would indicate a partially compromised immune system [117]. Decreased EGF values in children with ASD were also found in a study by Onore et al., reporting EGF levels three times lower, and by Tonhajzerova in a more recent study [85,110]. A different result was obtained from the study conducted by Pardo et al., analyzing serum (from children with autism and healthy controls) and cerebrospinal fluid (from children with autism), which demonstrated that EGF levels were significantly elevated in the serum of children with ASD. This could be interpreted as a suggestion that dysregulation of growth and modulation pathways may occur in autism [118]. This result was consistent with the findings from the study conducted by Tobiasova, in which cytokine levels in subjects with ASD were compared at baseline and after 8 weeks of treatment with Risperidone or placebo, showing an increase in EGF levels in subjects with ASD. The tests did not find significant differences between cytokine levels at baseline and after 8 weeks of treatment, and there was no association with clinical severity. In this same study, a significant correlation was found between EGF and MCP-1, a marker of chronic inflammation [115]. The hypothesis that EGF may play a role in the pathophysiology of autism was also considered in a study conducted by Işeri et al., which demonstrated that serum levels in children with ASD were significantly higher, with no correlation with clinical symptoms [116]. Currently, we find only one study in the literature that examined EGF levels in urine, finding no differences between the ASD group and the control group [94]. Aylward et al. found that children with autism aged 12 years or younger had significantly larger brain volumes compared to controls. However, there was no difference in those older than 12 years, suggesting that there is a slight decrease in brain volume in children starting from adolescence. High levels of EGF in children could contribute to accelerated postnatal brain growth in autism [18,94]. Studies suggest that EGF deficiency is persistent throughout the course of ASD [85]; in other cases, it has been hypothesized that EGF levels may increase at an early age and begin to decrease with advancing age [116]. Therefore, the literature appears inconsistent considering that no single marker seems to be consistently abnormal across studies [117].

### 3.4. Neuregulin (NRG)

NRGs are a group of proteins belonging to the EGF family that include several subtypes: NRG1, NRG2, NRG3, and NRG4. Notably, NRG1, NRG2, and NRG3 are expressed in the CNS. In the CNS, they interact with transmembrane tyrosine kinase receptors of the ErbB family, initiating intracellular signaling cascades, such as PI3K/Akt and MAPK/Erk. This cascade triggers different cellular responses including proliferation, migration, differentiation, and regulation of survival or apoptosis [119,120].

NRG1 is the most extensively studied member of the family, and it is known to perform a broad range of functions in many organs, including the heart, breast, and nervous system. Presumably, this versatility may be explained by the use of distinct 5′ flanking regulatory elements and alternative splicing. NRG1 generates six types of protein (I−VI) and many different isoforms, including heregulin, acetylcholine receptor-inducing activity (ARIA), and glial growth factor (GGF), each with distinct structures, tissue-specific expression patterns, and biological activities [120,121,122]. In the adult cerebral cortex, the most abundant NRG1 splicing variant is type III, followed by type II and type I. Specifically, NRG1 type I is crucial for adaptive neural responses essential for proper brain function and cognitive processes, as it regulates neuronal plasticity and neurotransmitter receptor expression with its activity-dependent regulation, enabling adaptive neural responses essential for proper brain function and cognitive processes. NRG1 type II, also known as GGF, plays a pivotal role in the differentiation and migration of radial glial cells during cerebral cortex development. Meanwhile, NRG1 type III is essential for various functions including the early development and survival of Schwann cells, sensorimotor gating working memory, promotion of oligodendrocyte myelination, and modulation of electrophysiological activity in the nucleus accumbens induced by the ventral hippocampus. These roles suggest that NRG1 type III may be involved in psychiatric disorders. In fact, studies on schizophrenia have identified several polymorphisms in the genes encoding NRG1 and its receptor ErbB4, providing a useful starting point for better understanding the pathogenic mechanisms of this disease [119,122]. Data from the literature proposed a possible implication of neuregulins in the etiopathogenic mechanisms of ASD. Indeed, NRG1 signaling has been suggested to play a role in the pathogenesis of autism, with evidence indicating an abnormal activation of these pathways. However, the conflicting and limited information has not yet explained the precise mechanism by which NRG1 contributes to the progression of autism (see Table 8) [123].

Ikawa et al. investigated the major NRG1 splicing variants (types I–VI) and observed a notable increase in the level of NRG1 type III in the peripheral blood mononuclear cells of children with ASD. This increase significantly correlated with the social interaction subscales of the Autism Diagnostic Interview-Revised (ADI-R), suggesting that NRG1 expression, particularly in microglia, may play a role in the pathophysiology of psychiatric disorders [119]. A further increase in the levels of NRGs in patients with ASD was measured by Esnafoglu in 2018, but no correlation was found with autistic symptoms [124].

Furthermore, Abbasy et al. conducted a case–control study on a pediatric cohort of 1540 ASD patients and 1490 neurotypical subjects, investigating the relationship between NRG1 levels and three domains of executive function (working memory, response inhibition, and vigilance), using blood samples. They found a significant correlation between a global reduction in all three types of NRG1 levels and deficiencies in executive functions in ASD patients, suggesting a potential involvement of IGF1, especially in response inhibition, vigilance, and working memory [121].

### 3.5. Fibroblast Growth Factor (FGF)

FGFs are a class of growth factors that can be divided into seven subfamilies based on sequence phylogeny. They are known to play various roles in the control of cell behavior [125,126]. In the central nervous system, growth factors such as FGF play a key role in brain plasticity and development. Studies reveal that FGF, implicated in cortical thickness and connectivity, could be responsible for neurodevelopmental disorders such as autism [127]. Based on this and the known neuroanatomical features of ASD, it has been suggested that a defect in FGF signaling, including alterations in its receptor, might play a role in the neuropathological mechanism underlying ASD. The same authors have hypothesized that mutations in genes regulating the FGF family may lead to autistic sensory hyper-responsiveness and be implicated in the etiopathogenesis of ASD [128,129]. FGF2 has been shown to be able to cross the blood–brain barrier in animal studies. Thus, serum levels of FGF2 may reflect brain levels of FGF2 [130,131]. The implication of FGFs in ASD has been studied in the literature (see Table 9).

Nour-Eldine et al. attempted to identify predictive biomarkers for early diagnosis of ASD by analyzing the plasma of 160 children (100 ASD and 60 controls) aged 2 to 4 years with no known concomitant clinical conditions with ASD and comparing it to a control group with negative familiarity for ASD. Among the cytokines examined, FGF2 was found to be significantly elevated in ASD compared with the control group. FGF2, along with eotaxin, HGF, and IFN-γ, was also shown to be the best predictor of ASD, with an overall accuracy of more than 80% [111]. An additional study was carried out in 2023 to investigate the serum levels of FGF2, IGF1, and VEGF in 40 children with ASD as the study group and 40 subjects with ASD and bipolar disorder (BD) as the control group. It was found that serum FGF2 levels were statistically significantly higher in the ASD + BD group. The study aimed to identify potential biomarkers for diagnosis and follow-up given the difficulty of the clinical management of BD in individuals with ASD and its prevalence in this population (5–7%) [97]. Esnafoglu also studied serum FGF2 levels in children with ASD. Sixty children with ASD and forty healthy children were involved. Serum levels of FGF2 in subjects with ASD were significantly lower than those in healthy control subjects. In addition, a negative correlation emerged between autistic symptoms evaluated with the CARS score and FGF2 for all subjects [132]. It is noteworthy that studies have shown that FGF dysregulation can create increased susceptibility to the development of ASD, contributing to the disruption of immune and neuronal functions associated with the disorder. ASD does not have a simple or single neuroanatomical phenotype that indicates obvious or consistent neurodevelopmental mechanisms. However, there is evidence of anatomical defects involving increased growth of the cerebral cortex, especially at the level of the frontal lobes, up to the third year of life and the size of the cerebellum in children younger than 5 years of age. The reason could be attributed to an increase in Fgf signaling [111,128,133]. By analyzing the mutations that reduce the expression of *Fgf8* and *Fgf17*, it was observed that these genes promote the growth of the cortex, and in particular the dorsomedial frontal cortex, which is the region found to be larger in ASD [134]. Several studies have attempted to identify reliable cytokine biomarkers for ASD; however, the results have been inconsistent. This could be due to differences in cohort characteristics and sample analysis approaches among different studies. Moreover, it is important to point out that the immune system undergoes dynamic changes during the developmental period, so some variability could be caused by comparing the results of studies performed on subjects of different age groups [111,135].

### 3.6. Hepatic Growth Factor (HGF)

HGF is a key factor that prevents neuronal death and promotes survival through pro-angiogenic, anti-inflammatory, and immunomodulatory mechanisms [97]. Recent evidence also suggests that HGF acts on neural stem cells to improve neuroregeneration [136]. The ability of HGF to modulate neuroinflammation is associated with an imbalance in CD4+ T cells, with pro-inflammatory (Th1 and Th17) and anti-inflammatory (Th2 and Tregs) subsets affecting dendritic cell function [137].

HGF and its tyrosine kinase receptor, encoded by the MET proto-oncogene, are expressed in the nervous system from prenatal development to adulthood, where they are involved in neuronal growth and survival. HGF-MET appears to be implicated in abnormalities in synaptogenesis underlying neurodevelopmental disorders such as ASD [137]. Table 10 summarizes the studies on the role of HGF in ASD.

The earliest studies on the role of HGF in the pathophysiology of autism date back almost two decades. In 2006, Sugihara et al. compared serum HGF levels in 17 high-functioning adult males with ASD and 18 neurotypical subjects, finding significantly reduced levels in the first group, but no clinical correlation [136].

Nour-Eldine et al. found significantly reduced levels of HGF in children with ASD compared to the controls. In particular, a combination of four cytokines (eotaxin, FGF2, HGF, and IFN-γ) was found to be the best predictor of ASD, with an overall accuracy greater than 80% [111].

A similar result was obtained by Russo et al. in 2013, who found that autistic children had significantly increased plasma levels of GABA and decreased levels of HGF [139]. While elevated GABA levels were associated with worsened impulsivity, stereotypies, and sensory abnormalities, reduced HGF levels showed no significant correlation with ASD symptom severity. Thus, a significant inverse relationship between plasma levels of HGF and GABA was found in autistic children [139].

HGF concentrations, along with EGF levels, were also assessed in another study conducted by Onore et al. involving 49 children with ASD and 31 age-matched controls with typical development. However, no significant differences were found in HGF levels between children with ASD and typically developing controls [85].

## 4. Conclusions

**Figure 2:**
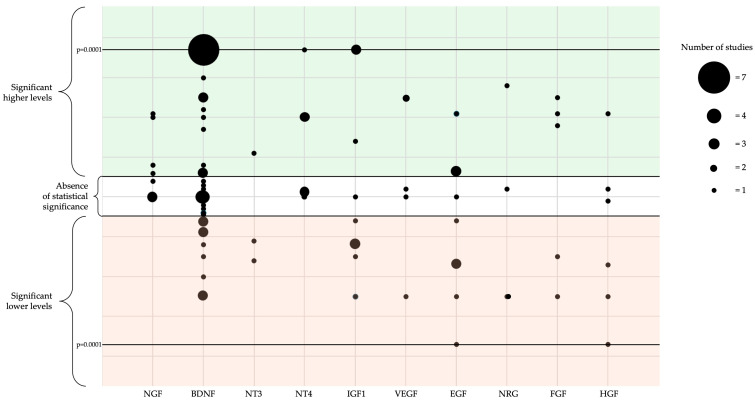
The figure summarizes the significance of the studies conducted on ASD and growth factors (GFs). Within the white area are the studies that did not reveal a statistically significant difference between GF levels in ASD patients and typically developed controls. In the green area (upper figure) are the studies that found significantly higher levels of GFs, while in the red area (lower figure) are the studies that found significantly reduced levels of GFs in ASD populations. The area of the dots represents the number of studies that found such significance, ranging from a minimum of one to a maximum of seven studies.

In conclusion, the involvement of neurotrophins and growth factors in ASD is complex, with both supportive and contradictory evidence in the literature. Neurotrophins such as NGF, BDNF, NT3, and NT4 play crucial roles in neuronal survival, synaptic plasticity, and brain development [19]. Neurotrophic signaling dysfunction, particularly involving BDNF, may involve microglial and mast cell activation, which, along with brain inflammation, are thought to contribute to ASD pathogenesis, possibly affecting neuronal connectivity [140,141]. Alterations in neurotrophin levels have been associated with ASD, suggesting potential links to cognitive deficits, sensory processing abnormalities, and behavioral issues. Similarly, growth factors like IGF1, VEGF, EGF, NRG, FGF, and HGF are implicated in neurogenesis, angiogenesis, and neural function regulation, with their altered levels also showing connections with ASD. However, many studies present inconsistent findings, which may be attributed to differences in research methodologies, including variations in sample sizes, patient populations, and diagnostic criteria (see Figure 2).

Discrepancies also arise from the use of different biological samples (i.e., serum, plasma, urine, CSF, brain tissue) and the influence of comorbid conditions, such as other neurodevelopmental disorders. One of the main challenges in studying growth factors in ASD is the heterogeneity in methodologies, including differences in biological samples such as serum, plasma, and cerebrospinal fluid. These variations can lead to inconsistent findings, as peripheral levels may not always reflect central nervous system activity. Future studies should prioritize standardized protocols for sample collection and analysis to enhance reproducibility and comparability across research efforts. Furthermore, the genetic and epigenetic conditions of the analyzed patients are often poorly defined, which can introduce additional variability and complicate the interpretation of results. These factors contribute to the challenges in drawing definitive conclusions.

To strengthen the findings, it is essential to use consistent methodologies and replicate results across different studies. Larger and more homogeneous populations should be studied to further investigate the relationship between different levels of neurotrophins and growth factors in autism, taking into account confounding factors such as age or platelet-derived production (as in the case of BDNF). The identification of altered levels of growth factors in ASD not only enhances our understanding of its pathophysiology but also opens potential avenues for clinical translation. Future studies should explore whether modulating these factors—through pharmacological interventions, dietary supplements, or lifestyle modifications—could offer targeted therapeutic benefits. Moreover, integrating growth factor analysis into early diagnostic protocols may improve early detection and personalized treatment strategies for individuals with ASD. Additionally, these factors should be analyzed in biological samples that allow for their easy and immediate use in clinical practice, facilitating their potential as reliable biomarkers for diagnosis. However, many studies reported altered values of neurotrophins and growth factors, underlining their potential involvement in neurodevelopmental processes. Further investigation is needed to explore the precise mechanisms through which these factors contribute to ASD pathophysiology.

Despite these inconsistencies, the neurotrophins and growth factors discussed hold potential as biomarkers and therapeutic targets for ASD. In this context, some compounds (i.e., supplementation with probiotics or flavonoids) have been proposed as potential therapeutic agents due to their ability to modulate neuroinflammation, enhance neurogenesis and neurotrophic signaling, and promote the expression of neurotrophins such as BDNF, NT3, and NGF [140,142,143,144].

Future research, with standardized methodologies, larger and more homogeneous cohorts, and a better definition of genetic and epigenetic factors, is needed to clarify their precise roles and evaluate their clinical relevance in ASD diagnosis and treatment.

## Figures and Tables

**Table 1:** Data from studies evaluating NGF levels in ASD subjects.

Reference	Population	Biological Sample	Results	Association with ASD Features
Nelson et al., 2001 [33]	69 ASD vs. 60 ID vs. 63 CP vs. 54 TD children	Neonatal dried blood spot samples	No significant difference in NGF	-
Dinçel et al., 2013 [28]	49 ASD vs. 49 TD children	Serum	↑ NGF	-
Rodrigues et al., 2014 [34]	30 ASD vs. 19 TD children	Plasma	No significant difference in NGF	-
Gomez-Fernandez et al., 2018 [35]	54 ASD vs. 54 TD children	Plasma	↑ NGF in ASD without regression	↑ NGF was found in the group of ANMR. This difference with the AMR subgroup could be linked to synaptogenesis.
Moradi et al., 2018 [36]	48 ASD children	Serum	↑ NGF	↑ NGF in ASD patients treated with a combined approach (perceptual–motor activities and vitamin D supplementation)
Mostafa et al., 2021 [32]	22 ASD vs. 22 TD children	Serum	↑ NGF	-
Gevezova et al., 2022 [31]	40 ASD vs. 12 TD children	Plasma	↑ NGF (correlation with parameters of MR)	-

Legend: ↑ = increased levels; AMR = ASD children with neurodevelopmental regression; ANMR = ASD children without neurodevelopmental regression; ASD = autism spectrum disorder; CP = cerebral palsy; ID = intellectual disability; MR = mitochondrial respiration; NGF = nerve growth factor; TD = typically developed; vs. = versus.

**Table 2:** Data from studies evaluating BDNF levels in ASD subjects.

Reference	Population	Biological Sample	Results	Association with ASD Features
Nelson et al., 2001 [33]	69 ASD vs. 60 ID vs. 63 CP vs. 54 TD children	Neonatal dried blood spot samples	↑ BDNF in ASD and ID	-
Miyazaki et al., 2004 [43]	18 ASD vs. 20 ID vs.16 TD children	Serum	↑ BDNF in ASD and ID	-
Connolly et al., 2006 [44]	48 autistic, 19 CDD, 14 PDD-NOS vs. 35 TD children	Serum	↑ BDNF	-
Nelson et al., 2006 [45]	47 ASD vs. 46 DS vs. 90 controls (28 very preterm infants, 50 term infants, and 12 adults)	Neonatal dried blood spot samples	No significant difference in BDNF	-
Hashimoto et al., 2006 [46]	18 male ASD vs. 18 age-matched male TD subjects	Serum	↓ BDNF	No significant correlation with ASD symptoms (ADI-R scores) and IQ levels
Katoh-Semba et al., 2007 [47]	56 ASD vs. 218 control subjects	Serum	Variable levels of BDNF at different ages (delayed developmental peak)	-
Croen et al., 2008 [48]	84 ASD vs. 49 children with ID vs. 159 TD controls	Neonatal dried blood spot samples	No significant difference in BDNF	-
Correia et al., 2010 [49]	146 ASD vs. 50 TD children	Platelet-rich plasma	↑ BDNF	No significant correlation with developmental level (GMDS-II quotients) and ASD severity (CARS scores)
Sheikh et al., 2010 [50]	7 ASD vs. 7 age-matched controls	Brain tissue (frontal cerebral cortex homogenate)	↓ BDNF	-
Makkonen et al., 2011 [51]	13 ASD vs. 15 TD children	Whole blood	No significant difference in BDNF	-
Ray et al., 2011 [52]	39 ASD vs. 18 TD control subjects	Plasma	↓ BDNF in severe ASD No significant difference in mild to moderate ASD	-
Al-Ayadhi, 2012 [53]	44 ASD vs. 40 age- and gender-matched TD children	Serum	↑ BDNF in mild ASD but not in severe ASD	-
Abdallah et al., 2013 [54]	359 ASD vs. 741 frequency-matched controls	Neonatal dried blood spot samples	Non-significant ↓ BDNF	-
Ricci et al., 2013 [55]	29 ASD vs. 29 age- and gender-matched TD subjects	Serum	↑ BDNF	Non-significant positive association with ASD severity
Kasarpalkar et al., 2014 [56]	48 ASD vs. 28 age-matched TD children	Serum	↑ BDNF in atypical ASD and ↓ BDNF in typical autistic females (including Rett syndrome)	-
Rodrigues et al., 2014 [34]	30 ASD vs. 19 TD children	Plasma	No significant difference in BDNF	-
Taurines et al., 2014 [57]	24 ASD vs. 20 age- and gender-matched TD children and adolescents	Serum	↓ BDNF Non-significant ↑ BDNF in ASD with ADHD than in ASD without ADHD	-
Zhang et al., 2014 [58]	60 ASD vs. 60 age- and gender-matched TD children	Serum	↑ BDNF	Significant positive association with ASD severity (CARS scores)
Bryn et al., 2015 [59]	65 ASD vs. 30 age- and gender-matched TD children	Plasma	↑ BDNF No significant difference in proBDNF	-
Halepoto et al., 2015 [60]	60 ASD vs. 25 age- and gender-matched TD children	Serum	↑ BDNF	-
Wang et al., 2015 [61]	75 ASD vs. 75 age- and gender-matched TD children	Serum	↑ BDNF	Significant positive association with ASD severity (CARS scores)
Meng et al., 2016 [62]	82 ASD vs. 82 age- and gender-matched TD children	Serum	↑ BDNF	Significant positive association with ASD severity (CARS scores) Significant negative association with IQ levels
Francis et al., 2018 [64]	45 ASD vs. 26 age-matched TD children	Serum	↓ BDNF	No significant correlation with ASD severity (CARS and ADOS-2 scores), ASD type, or adaptative functioning (VABS scores)
Gomez-Fernandez et al., 2018 [35]	54 ASD vs. 54 TD children	Plasma	No significant difference in BDNF	-
Ormstad et al., 2018 [65]	65 ASD vs. 30 TD children	Serum, plasma	↑ BDNF ↑ BDNF in ASD with ID than in controls and ASD without ID	-
Ghafouri-Fard et al., 2019 [66]	50 ASD and 50 age- and gender-matched TD children	Whole blood	↑ BDNF	-
Skogstrand et al., 2019 [67]	801 ASD vs. 2421 controls	Neonatal dried blood spot samples	↓ BDNF	-
Barbosa et al., 2020 [68]	49 ASD vs. 37 TD children	Serum	↑ BDNF	-
Bozkurt et al., 2021 [69]	33 ASD vs. 27 age-matched TD children	Serum	↑ BDNF	No significant correlation with regressive type of ASD
Farmer et al., 2021 [70]	94 ASD vs. 21 DD vs. 52 TD children	Serum	↑ BDNF in ASD and DD	-
Robinson-Agramonte at al., 2022 [71]	22 ASD vs. 29 TD children	Serum	No significant difference in BDNF ↓ proBDNF	No significant correlation with ASD severity (CARS scores)
Han et al., 2022 [72]	21 ASD vs. 19 TD children	Serum	↓ BDNF	Significant negative correlation with social interaction, communication, and stereotyped behavior subscales of the ADI-R
Carpita et al., 2024 [73]	22 ASD vs. 22 TD adult subjects	Platelets	↓ platelet BDNF	Significant negative correlation with ASD severity (AdASS scores)
Cui et al., 2024 [74]	67 ASD vs. 47 ID subjects	Serum	No significant difference in BDNF ↓ proBDNF ↑ mBDNF/proBDNF	Significant positive correlation between BDNF and ASD severity (CARS scores)

Legend: ↓ = decreased levels; ↑ = increased levels; AdASS = Adult Autism Subthreshold Spectrum; ADHD = attention deficit/hyperactivity disorder; ADI-R = Autism Diagnostic Interview-Revised; ADOS-2 = Autism Diagnostic Observation Schedule 2; ASD = autism spectrum disorder; BDNF = brain-derived neurotrophic factor; CARS = Childhood Autism Rating Scale; CDD = childhood disintegrative disorder; CP = cerebral palsy; DD = developmental delay; DS = Down syndrome; GMDS-II = Griffiths Mental Developmental Scale II; ID = intellectual disability; IQ = intelligence quotient; mBDNF = mature brain-derived neurotrophic factor; TD = typically developed; VABS = Vineland Adaptive Behavioral Scales; vs. = versus.

**Table 3:** Data from studies evaluating NT3 levels in ASD subjects.

Reference	Population	Biological Sample	Results	Association with ASD Features
Nelson et al., 2001 [33]	69 ASD vs. 60 ID vs. 63 CP vs. 54 control subjects	Neonatal dried blood spot samples	No significant difference in NT3	-
Nelson et al., 2006 [45]	47 ASD vs. 46 DS vs. 90 controls (28 very preterm infants, 50 term infants, and 12 adults)	Neonatal dried blood spot samples	↓ NT3	-
Sajdel-Sulkowska et al., 2009 [79]	8 ASD vs. 7 ASD	Cerebellar	↑ NT3	-
Tostes et al., 2012 [78]	24 ASD vs. 24 TD	Plasma	↓ NT3	
Rodrigues et al., 2014 [34]	30 ASD vs. 19 TD children	Plasma	No significant difference in NT3	-
Segura et al., 2015 [80]	10 ASD vs. 21 TD children	Blood	↓ NT3	-

Legend: ↓ = decreased levels; ↑ = increased levels; ASD = autism spectrum disorder; CP = cerebral palsy; DS = Down syndrome; ID = intellectual disability; TD = typically developed; NT3 = neurotrophin-3; vs. = versus.

**Table 4:** Data from studies evaluating NT4 levels in ASD subjects.

Reference	Population	Biological Sample	Results	Association with ASD Features
Nelson et al., 2001 [33]	69 ASD vs. 60 ID vs. 63 CP vs. 54 control subjects	Neonatal dried blood spot samples	↑ NT4 in ASD and ID than in CP and controls	-
Miyazaki et al., 2004 [43]	18 ASD vs. 20 ID vs. 16 control subjects	Serum	No significant difference in NT4	-
Nelson et al., 2006 [45]	47 ASD vs. 46 DS vs. 90 controls (28 very preterm infants, 50 term infants, and 12 adults)	Neonatal dried blood spot samples	No significant difference in NT4	-
Abdallah et al., 2013 [54]	359 ASD vs. 741 frequency-matched controls	Neonatal dried blood spot samples	↓ NT4	-
Rodrigues et al., 2014 [34]	30 ASD vs. 19 TD children	Plasma	No significant difference in NT4	-
Segura et al., 2015 [80]	10 ASD vs. 21 TD children	Blood	↓ NT4	-

Legend: ↓ = decreased levels; ↑ = increased levels; ASD = autism spectrum disorder; CP = cerebral palsy; DS = Down syndrome; ID = intellectual disability; TD = typically developed; NT4 = neurotrophin-4; vs. = versus.

**Table 5:** Data from studies evaluating IGF1 levels in ASD subjects.

Reference	Population	Biological Sample	Results	Association with ASD Features
Vanhala et al., 2001 [90]	11 ASD vs. 11 TD children	CSF	↓ IGF1	No significant correlations between IGF1 and head circumferences
Vargas et al., 2005 [91]	6 ASD vs. 9 TD children	CSF	↑ IGFBP3 ↑ IGFBP4	-
	15 ASD vs. 12 TD children and adults	Brain tissue	↑ IGF1 levels in the ante-rior cingulate gyrus	
Riikonen et al., 2006 [92]	25 ASD vs. 16 TD children	CSF	No difference in IGF2 ↓ IGF1	Significant negative correlation between IGF1 and head circumference
Mills et al., 2007 [93]	71 ASD vs. 59 TD children	Plasma	↑ IGF1	Significant positive correlation between IGF1 and head circumference
Anlar et al., 2007 [94]	34 ASD vs. 29 TD children	Urine	↓ IGF1	-
Cioana et al., 2020 [95]	15 ASD vs. 20 TD children and adults	Brain tissue	No significant differences in IGF1 protein or mRNA levels in fusiform gyrus tissues between ASD and controls	-
Şimşek et al., 2021 [84]	40 ASD vs. 40 TD children	Serum	↑ IGF1	No significant correlation between IGF1 and sociodemographic and clinical scale scores
Robinson-Agramonte at al., 2022 [71]	22 ASD vs. 29 TD children	Serum	↑ IGF1	Significant positive correlation between CARS scores and IGF1 levels
Li et al., 2022 [96]	150 ASD vs. 165 TD children	Serum	The children with severe ASD had significantly ↓ IGF1 and IGFBP3 than those with mild to moderate ASD	Significant negative correlation between IGF1 and the total score of CARS
Guldiken et al., 2023 [97]	40 ASD vs. 40 TD children	Serum	No difference in IGF1	-

Legend: ↓ = decreased levels; ↑ = increased levels; ASD = autism spectrum disorder; CARS = Childhood Autism Rating Scale; CSF = cerebrospinal fluid; IGF1 = insulin-like growth factor 1; IGF2 = insulin-like growth factor 2; IGFBP3 = insulin-like growth factor-binding protein-3; IGFBP4 = insulin-like growth factor-binding protein-4; TD = typically developed; vs. = versus.

**Table 6:** Data from studies evaluating VEGF levels in ASD subjects.

Reference	Population	Biological Sample	Results	Association with ASD Features
Vargas et al., 2005 [91]	6 ASD vs. 9 TD children	CSF	↑ VEGF	**-**
Emanuele et al., 2010 [107]	22 ASD vs. 28 TD adult	Serum	↓ VEGF ↑ sVEGFR1	-
Kajizuka et al., 2010 [108]	31 ASD vs. 31 TD male children	Serum	No difference in VEGF	-
Zakareia et al., 2012 [109]	40 ASD vs. 20 TS	Plasma	No difference in VEGF	-
Skogstrand et al., 2019 [67]	801 ASD vs. 2421 controls	Neonatal dried blood spot samples	No difference in VEGF-A	-
Şimşek et al., 2021 [84]	40 ASD vs. 40 TD children	Serum	No difference in VEGF-A	-
Tonhajzerova et al., 2021 [110]	25 ASD vs. 25 TD children	Plasma	No difference in VEGF	-
Guldiken et al., 2023 [97]	40 ASD vs. 40 TD children	Serum	↑ VEGF	-
Nour-Eldine et al., 2024 [111]	100 ASD vs. 60 TD children	Plasma	No difference in VEGFA	-

Legend: ↓ = decreased levels; ↑ = increased levels; ASD = autism spectrum disorder; CSF = cerebrospinal fluid; sVEGFR1 = soluble vascular endothelial growth factor receptor-1; TD = typically developed; VEGF = vascular endothelial growth factor; VEGFA = vascular endothelial growth factor A; vs. = versus.

**Table 7:** Data from studies evaluating EGF levels in ASD subjects.

Reference	Population	Biological Sample	Results	Association with ASD Features
Suzuki et al., 2007 [114]	17 ASD vs. 18 TD adults	Serum	↓ EGF	-
Anlar et al., 2007 [94]	34 ASD vs. 29 TD children	Urine	No difference in EGF	-
Tobiasova et al., 2011 [115]	77 ASD vs. 19 TD children	Serum	↑ EGF	-
Iseri et al., 2011 [116]	27 ASD vs. 28 TD children	Serum	↑ EGF	-
Manzardo et al., 2012 [117]	99 ASD vs. 40 TD children	Plasma	↓ EGF	-
Onore et al., 2012 [85]	49 ASD vs. 31 TD children	Plasma	↓ EGF	-
Russo, 2013 [113]	49 ASD (11 PPD-NOS) vs. 40 TD children	Plasma	↓ EGF	Significant negative correlation between EGF and symptoms: hyperactivity, general motor skills, and digitigrade walking
Pardo et al., 2017 [118]	104 ASD vs. 54 TD children	Serum	↑ EGF	-
Tonhajzerova et al., 2021 [110]	25 ASD vs. 25 TD children	Plasma	↓ EGF	-

Legend: ↓ = decreased levels; ↑ = increased levels; ASD = autism spectrum disorder; EGF = epidermal growth factor; PPD-NOS = pervasive developmental disorder-not otherwise specified; TD = typically developed; vs. = versus.

**Table 8:** Data from studies evaluating NRG levels in ASD subjects.

Reference	Population	Biological Sample	Results	Association with ASD Features
Ikawa et al., 2017 [119]	29 ASD vs. 30 TD children	Peripheral blood mononuclear cells	↑ NRG1 type III	Positive correlation between NRG1 type III and social interaction subscales of the ADI-R No significant correlations between NRG1 type III and communication, stereotyped behavior subscales of the ADI-R
Esnafoglu, 2018 [124]	32 ASD vs. 32 TD children	Serum	↑ NRG1	No significant correlations identified between NRG1 and autistic symptoms (CARS scores)
Abbasy et al., 2018 [121]	1540 ASD vs. 1490 TD children	Blood	↓ NRG1 type I ↓ NRG1 type II ↓ NRG1 type III	Significant correlation between ↓ NRG1 type I and type III and lower performance of all IVA tests Significant correlation between ↓ NRG1 type II and lower performance of VRCQ Significant correlation between ↓ NRG1 type II and higher score of ADOS

Legend: ↓ = decreased levels; ↑ = increased levels; ADI-R = Autism Diagnostic Interview-Revised; ADOS = Autism Diagnostic Observation Schedule; ASD = autism spectrum disorder; CARS = Childhood Autism Rating Scale; IVA = Integrated Visual and Auditory; NRG1 = neuregulin 1; TD = typically developed; VRCQ = Visual Response Control Quotient; vs. = versus.

**Table 9:** Data from studies evaluating FGF levels in ASD subjects.

Reference	Population	Biological Sample	Results	Association with ASD Features
Vargas et al., 2005 [91]	6 ASD vs. 9 TD children	CSF	↑ FGF4 ↑ FGF9	-
Esnafoglu and Ayyıldız, 2017 [132]	60 ASD vs. 40 TD children	Serum	↓ FGF2	Significant negative correlation between FGF2 and CARS score
Guldiken et al., 2023 [97]	40 ASD vs. 40 TD children	Serum	↑ FGF2	-
Nour-Eldine et al., 2024 [111]	100 ASD vs. 60 TD children	Plasma	↑ FGF2	-

Legend: ↓ = decreased levels; ↑ = increased levels; ASD = autism spectrum disorder; CARS = Childhood Autism Rating Scale; CSF = cerebrospinal fluid; FGF2 = fibroblast growth factor 2; FGF4 = fibroblast growth factor 4; FGF9 = fibroblast growth factor 9; TD = typically developed; vs. = versus.

**Table 10:** Data from studies evaluating HGF levels in ASD subjects.

Reference	Population	Biological Sample	Results	Association with ASD Features
Vargas et al., 2005 [91]	6 ASD vs. 9 TD children	CSF	↑ HGF	-
Sugihara et al., 2006 [136]	17 ASD vs. 18 TD adults	Serum	↓ HGF	-
Russo et al., 2009 [138]	29 ASD vs. 31 TD	Serum	↓ HGF	-
Onore et al., 2012 [85]	49 ASD vs. 31 TD children	Plasma	No difference in HGF	-
Gomez-Fernandez et al., 2018 [35]	54 ASD vs. 54 TD children	Plasma	No significant difference in HGF	-
Nour-Eldine et al., 2024 [111]	100 ASD vs. 60 TD children	Plasma	↓ HGF	-

Legend: ↓ = decreased levels; ↑ = increased levels; ASD = autism spectrum disorder; CSF = cerebrospinal fluid; HGF = hepatic growth factor; TD = typically developed; vs. = versus.
